# Possible Sources of Trace Metals in Obese Females Living in Informal Settlements near Industrial Sites around Gauteng, South Africa

**DOI:** 10.3390/ijerph20065133

**Published:** 2023-03-14

**Authors:** Gladness Nteboheng Lion, Joshua Oluwole Olowoyo

**Affiliations:** 1 Department of Biology and Environmental Sciences, Sefako Makgatho Health Sciences University, Pretoria 0204, South Africa; 2 Department of Health Science and The Water School, Florida Gulf Coast University, Fort Myers, FL 33965, USA

**Keywords:** trace metals, females, obesity, mining, coal fired power station

## Abstract

Trace metals have been reported in the literature to be associated with obesity. Exposure to some trace metals such as Mn, Cr, Ni, Cd, and Pb may pose a serious health risk to individuals living around a polluted environment. The present study assessed the levels of trace metals in the blood of obese females living around industrial areas in Gauteng, South Africa. The study was carried out using a mixed method approach. Only females with a BMI ≥ 30.0 were considered. A total of 120 obese females participated in the study (site 1: 40—industrial area, site 2: 40—industrial area, and site 3: 40—residential area), aged 18–45 and not in menopause. Blood samples were analysed for trace metals content using inductively coupled plasma mass spectrometry (ICP-MS). The mean concentrations of trace metals were in the order Pb > Mn > Cr > Co > As > Cd (site 1), Pb > Mn > Co > As > Cd (site 2), and Mn > Cr > Co > As > Pb > Cd (site 3). The blood Mn from site 1 ranged from 6.79 µg/L–33.99 µg/L, and the mean differences obtained from the participants from different sites were significant (*p* < 0.01). The blood levels of Mn, Pb, Cr, Co, As, and Cd were above the recommended limits set by the WHO in some of the participants. The present study noted, among others, closeness to industrial areas, lifestyle decisions such as the use of tobacco products by their partners indoors, and the method used for cooking as factors that might have accounted for the blood levels of Mn, Pb, Cd and Co. The study showed that there is a need for constant monitoring of the levels of trace metals in the blood of those living in these areas.

## 1. Introduction

Comorbidities of human exposure to pollutants such as trace metals have been linked to obesity with worse health consequences and more complex clinical management [1,2]. Individuals living with obesity are more susceptible to the harmful effects of environmental pollutants, including trace metals [3]. Obesity is a rapidly growing problem in the world and in South Africa [4]. In South Africa, a report from the Heart and Stroke Foundation South Africa [5] suggests that the percentage of obese individuals has increased from 2002 to present. A study conducted in 2015 by the University of North West School of Biokinetics, Recreation and Sport Science revealed that nearly two-thirds of the South African population is overweight. The study further revealed that 70 percent of females are overweight. Reports from the literature show that females are most at risk of developing diseases associated with obesity [6,7,8,9].

Trace metals are natural components of the ecosystem but are on the rise due to various developmental programs [10,11]. Natural sources of trace metals include weathering of rock material, soil erosion, volcanic activity, and dissolution of water-soluble salts [12], and they can be added to the environment through anthropogenic activities such as fertilizers, biosolids, irrigation water, coal combustion residues, mining, waste disposal, vehicular emissions, urban runoff, and emissions from industrial activities [13,14,15]. Exposure to trace metals such as Mn, Cr, Ni, Cd, and Pb as a result of industrial activities and vehicle emissions has become a great concern [16]. Trace metals contribute to a variety of diseases, aging, and neurological and behavioural disorders [17,18]. The negative impact of trace metals in human tissue has been reported in the literature, including neurotoxicity, haemolysis, cancer, and cardiomyopathy [19,20,21].

Human exposure to trace metals may occur in three different pathways, which include inhalation, ingestion and dermal exposure. Through the ingestion pathway, trace metals can be transferred into food chains, where they can be absorbed by plants, which are consumed by humans [22]. Street et al. [23] noted in South Africa that informal backyard foundries might increase the likelihood of exposure to toxic metals for workers, family members, and communities. The study by Lion and Olowoyo [24] showed that agricultural soils may contain high amounts of trace metals, which may be bioaccumulated by plants. Exposure to trace metals via inhalation or dermal contact has also been documented. In a separate study conducted by Lion and Olowoyo [25], exposure to trace metals such as Cr, Cd, and As were documented. The procedure followed the use of the Target Hazard Quotient, which was first used by Nriagu et al. [26]. The study showed that the population around the area under investigation may be exposed to these toxic trace metals through inhalation or dermal exposure. In a study that compared trace metals in the blood of occupationally exposed mine workers and non-occupationally exposed individuals living around a mining area, it was reported that the trace metals Mn, Cd, As, and Pb were higher in the occupationally exposed group [27]. Exposure to trace metals such as Mn, Fe, Al, and Pb has been linked to Alzheimer’s disease [28,29,30]. In another study conducted by Etsuyankpa et al. [31] on occupationally exposed workers, the results showed that urine samples had higher concentrations of Mn, Pb, and Ni, while in blood samples, there were high concentrations of Mn, Pb, Ni, and Cr. The concentration of Mn in the blood samples ranged from 14.22 to 22.63 µg/L, and this concentration ranged from 8.30 to 12.20 µg/L in the urine. The concentration of Pb in blood varied between 190.10 and 212.10 µg/L, while the concentration in urine varied from 141.90 to 215.00 µg/L. Additionally, prolonged human exposure to Pb has been reported to decrease performance in the nervous system; cause weakness in the wrists, fingers and ankles; and increase blood pressure and anemia [32]. In South Africa, Nkosi et al. [33] reported that those living around mining areas have high levels of blood pressure. The study of Zunpuski et al. [34] further showed that children and adults living around gold mine tailings may have detectable amounts of uranium with higher concentrations in children’s hair when compared to adults.

Mining activities have been reported to have adverse effects on the environment and human health [35,36]. Kamunda et al. [37] showed that the concentrations of Cr and Ni in soils collected around a gold mine basin in the Witwatersrand were higher than the permissible limit and further indicated a carcinogenic health risk for humans living around the area. A study by Olowoyo and Lion [38] had previously reported that people living in areas exposed to environmental pollution, such as those around mining and coal-fired power stations, may suffer from respiratory diseases because of pollutants around these areas. A similar study conducted by Okereafor et al. [39] also showed that trace metals such as Mn, Cr, Pb, Ni, Fe, and Cd were major pollutants from mining areas. The challenge and great dilemma are that mining contributes a great portion to the economy of South Africa. Mining alone contributes an income of USD 588 billion and is responsible for the employment of more than 451,427 people [35,40]. However, some of these mines are open cast, and it is common to see heaps of uncovered excavated waste material from mining sites in close proximity to informal settlements [38]. The pollutants from underground end up on the surface of the ground and are carried by the wind as dust to the residents living around the mining site in the informal settlements.

Apart from mining, activities such as coal-fired power stations have also been reported as contributors of trace metals in the environment. Coal-fired power stations may expose the surrounding communities to pollutants that may have a negative impact on their health [38]. The impact of human exposure to coal combustion varies according to the composition of the coal. The study by Olowoyo et al. [26] conducted around a coal-powered station in Pretoria, South Africa, showed that plants and soil collected around this area had elevated levels of trace metals both in the soil and plants. Previous studies noted that the burning of coal from Brazil releases high levels of Zn and As [41,42], while coal-fired power stations in South Africa release high levels of Pb in the environment [43].

Over the years, South Africa has been under great pressure to sustain the increasing need for energy in the country. The country’s electric power supplier relies on the combustion of coal for the generation of electricity to fuel the country [44]. Coal is a fossil fuel and non-renewable energy source that is combusted and used to generate electricity [45]. A coal-fired power plant is among the great generators of environmental pollution, releasing large quantities of pollutants which include aerosol. As the status of load shedding and incessant power failures experienced in South Africa continues, the constant burning of coal may also increase the level of pollution in the environment. Eskom has also resorted to the burning of diesel, a form of fuel that has been reported to increase high levels of trace metals such as Fe, Cu, Cr, Ti and Mn in the environment [46].

Recent reports showed an association between trace metals and health complications among obese individuals [47,48,49]. The impact of exposure to the trace metals As, Pb, Cr, and Zn on obesity development and cardiovascular disease in children and adolescents was investigated by Nasab et al. [50]. Their findings showed that exposure to these metals might be a risk factor for children and adolescents to develop cardiovascular disease and obesity markers. Furthermore, they also demonstrated that these metals were associated with individual weight status. They concluded that there was a significant association between trace metals (As, Pb, Cr, and Zn) and body mass index (BMI), waist circumference (WC), fasting blood sugar (FBS), and lipid profile, among other things. These metals have also been reported to have the ability to raise leptin levels in the body, which is a protein produced by adipose tissue. Studies relating to trace metals, female obesity, and its comorbidities have not been carried out in detail in populations exposed to mining, coal-fired power stations, and other industrial activities in South Africa.

Human exposure to pollutants such as Mn, Cr, Pb, and As due to living around a mining site and coal-fired power station may have negative health implications [48,51]. However, when coupled with obesity, the consequences maybe be fatal to humans [50,52]. The current study investigated the levels of trace metals in obese female individuals living in areas impacted by different areas close to industries. Though most of these areas are informal settlements, there are people living in these areas for different reasons. As stated above, mining and coal-fired power stations have the ability to introduce different types of trace metals into the environment; however, a comparison of the impact of these activities on humans and with particular reference to obese individuals living in areas associated with these types of industries has not been documented, and the effect is not fully known. This current study investigated the impact of these activities on the concentrations of trace metals in the blood of these individuals. To the best of our knowledge, this is the first study to compare and provide information on the impacts of different industries in introducing trace metals in the environment and may therefore serve as baseline data for future research. Hence, the aim of this study was to determine the concentration of trace metals in the blood of obese females and compare the impact of living around industrial sites and an area that was not industrial but instead residential.

## 2. Methodology

### 2.1. Study Design and Setting

The study was carried out using a mixed methods approach. The study was carried out around two industrial areas in Pretoria, and participants were obese females living in an informal settlement in these areas. A purposive sampling method was used for the study. As per observation from the first visit to the industrial areas, site 1 had 82 housing units, and site 2 had only 65 housing units. It was then assumed that there was an adult female member living in each house. According to the report provided by [53], 70% of women in SA are obese; hence it was envisaged that there were about 57 obese females from industrial site 1 and 46 obese individuals from industrial site 2. However, only 120 obese females participated in the study who were aged between ages 18–45 and had not reached menopause. The participants sampled were 40 obese females from site 1, 40 obese females living in site 2, and 40 obese females living far away from these two sites in a residential area.

### 2.2. Sampling and Study Population

Ethical clearance was obtained prior to the commencement of this study. Approval was obtained from Sefako Makgatho University Research Ethics Committee (SMUREC), with reference number SMUREC/S/39/2018:PG. A written informed consent was issued to each willing participant. Owing to the sensitive nature of the study, the identity of human subjects was protected. No unique identifiers were collected, and the study was conducted with only self-selected participants. The study was part of a Ph.D. program of the leading author and, as such, a cohort study. The authors had initially examined the relationship between the trace metal levels and reproductive hormones from some participants in this study [54].

The study only included obese individuals living around the industrial sites and a group from a residential area from a different site not impacted by industrial activities. Females with a higher BMI (≥30.0) were used as obese participants in the study, and those with a lesser BMI (≤29.9) were excluded. Individuals that were taking mineral supplements and those diagnosed with diabetes were also excluded from the study. Diabetic individuals within these groups were excluded from the study due to the fact that their condition may eliminate trace metals through the urine, and therefore, the results may be compromised [51]. The blood samples from all obese female participants were drawn by puncturing a vein. The procedure was performed by a qualified professional under controlled conditions to minimize contaminations. Only 50 mL of blood samples were collected using the certified BD Vacutainer sterile tubes for trace metals. An anticoagulant of 143 USP units of sodium heparin was added to each tube and enclosed with a royal blue hemoguard cap. Samples were then stored at cool temperatures before being analyzed.

### 2.3. Trace Metal Determination

The analysis of blood samples for trace metals determination was carried out at accredited laboratories. An inductively coupled plasma mass spectrometer (ICP–MS) Thermo (Bremen, Germany) X-Series 2, with a concentric glass nebulizer and Peltier-cooled glass spray chamber, were used to analyse trace metals from the blood samples collected from all sites at an accredited lab. From the samples, 100µL of blood sample was diluted 45-fold with milli-Q water (>18 megohm/cm resistivity) containing 1% nitric acid (Optima grade, Fisher Scientific, Shanghai, China) and 0.01% TritonX-100 and allowed to digest overnight at room temperature. Then, 500µL hydrogen peroxide (30% Suprapur grade, Sigma-Aldrich, St. Louis, MI, USA) was added to each digest and allowed to sit for at least one hour prior to analysis. Then, the digested solution was diluted with distilled water and brought up to 50 mL volume. The blanks were prepared by adding reagents to deionized water in place of the samples so as to monitor the background concentration of all the analytes.

### 2.4. Data Analysis

SPSS 26.0 for windows was used to carry out the statistical analysis. Analysis of variance was used to test for significant differences in the concentrations of trace metals in the blood of obese female participants from all the sites.

### 2.5. Quality Control and Assurance

For quality control, all the materials, including the pipettes, glassware, and stoppers, were washed with 10% nitric acid and rinsed with distilled water. The certification for ICP-MS was obtained by calibrating the machine with certified reference material before introducing the blanks and the samples. For quality assurance, samples were analyzed in triplicates, and a single wash was performed after a complete analysis of each sample.

## 3. Results and Discussion

The participants in this study comprised only of females. The participants from sites 1 and 2 were all living in an informal settlement just around the industrial areas. Most of the participants were staying with their partners, who were casual workers in some of these industries. The age range was 18–45. From the study, 33% of the participants had been staying in site 1 for a period between 1 and ≥ 10 years, 33 % had been staying in site 2 for 1– ≥ 10 years, and the remaining group was the participants from a residential area. Within the residential area group, the information received later showed that some participants had resided in areas not far from industrial activities at site 1 for more than 10 years. Most of the participants mentioned that their partners were active smokers, and none of them were smokers. The mode of cooking at home involved the use of firewood and the burning of charcoals in most cases. These two activities, as mentioned by some of the participants, may increase the concentrations of trace metals such as Pb, Cd, and As in their blood. The study by Jung et al. [55] showed that passive smoking or exposure to secondhand smoke may increase blood Cd. El Mohr et al. [56] also reported that passive smoking, as noted in our study, may increase the levels not just of Cd but also Pb and As in the serum of passive smokers. The study by Olufunsho et al. [57] also noted that exposure to gaseous pollutants and dust, which may come in the form of ashes, may increase the levels of toxic trace metals in the blood. The above-mentioned phenomena were noticed and practiced on a daily basis by some of the participants who presented high levels of toxic trace metals in our study.

The results of trace metal analysis from blood samples collected from obese females from all the participants are represented in Table 1. From the results obtained from some participants, the mean concentrations were observed to be higher than the recommended limit by the WHO for human exposure to trace metals such as Cr, Co, As, and Pb. Furthermore, Cd concentrations were above recommended values by the WHO in some individuals that participated in the study, even though the mean concentration of Cd was lower than the recommended limit. Overall, the recorded mean concentrations of all trace metals in the study were in the order Mn > Pb > Cr > Co > As > Cd (site 1), Pb > Mn > Co > As > Cd (site 2), and Mn > Cr > Co > As > Pb > Cd (site 3).

**Figure 1:**
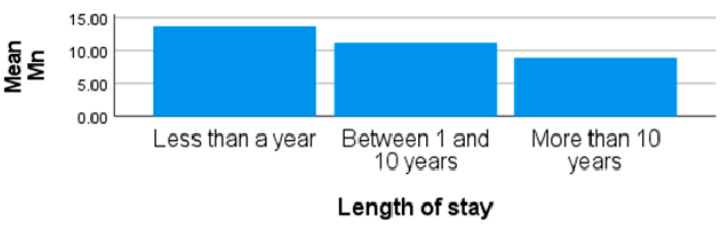
Showing blood Mn by length of stay in obese females living around site 3.

The highest mean concentration of Mn (12.97 ± 6.04 µg/L) from obese females in site 1 was significantly higher than other sites (*p* < 0.01). The second highest concentrations of Mn in obese female participants were recorded from site 2, with a mean and standard deviation of 11.41 µg/L and 4.35 and a range of 5.50–24.40 µg/L. The values recorded for Mn in the blood of some participants from all the sites were above the WHO recommended limit of 12.60 µg/L. There were significant differences in the concentrations of Mn from participants residing in site 1, site 2, and site 3 (*p* < 0.01). The lowest mean Mn concentrations in the participant’s blood were recorded from site 3. Although some participants live around site 3 for employment and study purposes, they frequently visit their homes which are either in sites 1 and 2, and these areas are associated with mining activities and other industrial activities. Due to the location of site 3 and nature of work of some participants in site 3, some are usually exposed to vehicular emissions on a daily basis, and the findings from this study should assist in having a basis for further research into the source of the Mn that was high in the blood of the participants in this area. The length of stay of obese females living around site 3 shows that individuals who lived in the area for more than 10 years recorded lower levels of blood Mn than those who have stayed in the area for less than 10 years (Figure 1). This suggests that those individuals may have been exposed to high levels of Mn in their previous residential areas [58]. The values of Mn recorded from site 1, an industrial area with active activities taking place from the industry situated opposite the informal settlement, are similar to those reported by Dey et al. [59], where the blood Mn from samples collected around a mining site exceeded the recommended limit. Their findings showed that Mn is considered a systemic toxicant that can damage multiple organs of humans. High levels of Mn may have adverse effect on the neurobehavioral attitude, as reported in the study by Pesch et al. [60]. The reports from other studies, such as Aguera et al. [61], showed mean values of 2.7 µg/L in females and 1.0 µg/L in males that are working as farmers and exposed to pesticides.

**Figure 2:**
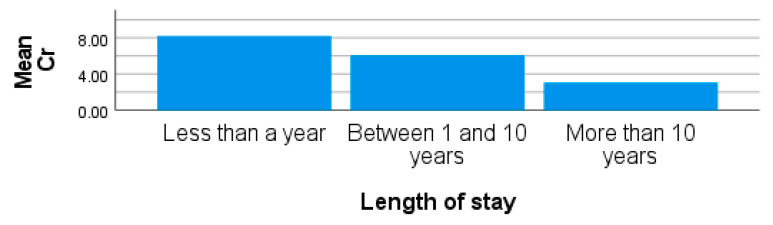
Showing blood Cr by length of stay in obese females living around site 3.

The mean concentration of Cr from site 3 was 6.16 ± 6.30 µg/l and in the range of 1.58–29.29 µg/L. From participants in site 1, Cr concentrations recorded a mean and standard deviation of 3.70 µg/L and 4.11 µg/L, respectively. The range for blood Cr was between 0.41 and 19.01 µg/L for the participants in the study from this site. Cr in blood from participants from site 2 was below the detection limit. The levels of Cr in the blood of participants may be as a result of emissions from industrial activities. Levels of blood Cr recorded for site 3 could be as previously indicated due to the participants’ previous area of occupation. Site 3 is characterized by an influx of individuals who previously lived in other areas, including places around industrial areas. The blood Cr could be historical traces of their previous and current living area since trace metals do not biodegrade but rather bio-accumulate (Figure 2). In the absence of any known exposure to induce the levels of blood Cr recorded from site 3, this study is therefore suggesting a source of significant Cr exposure in this site, which will require further research involving more participants. Increased levels of Cr may also be due to exposure to tobacco products, in this case through passive smoking [26,56]. Furthermore, the findings of this study were similar to that of Etsuyankpa et al. [30], where the concentration of Cr, Mn, Pb, and Cd in the blood exceeded the set recommended limit in both the occupationally exposed groups and non-exposed groups (control). High Cr levels have been associated with microcytic anaemia and mitochondrial and DNA damage in blood cells, which in turn induces carcinogenicity, occupational asthma, airway hypersensitivity, and nose, eye and skin irritation [62].

**Figure 3:**
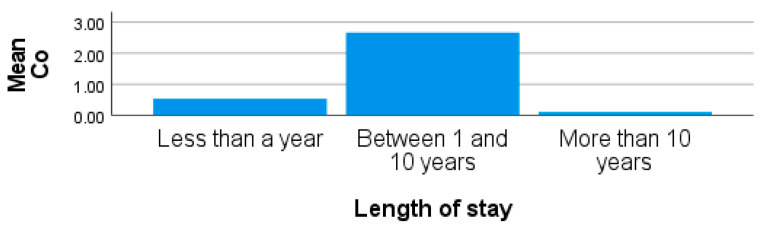
Showing blood Mn for length of stay in obese females living around site 3.

The concentration of Co from participants from site 3 was higher than the other two sites, with a mean and standard deviation of 2.40 ± 8.92 µg/L and with values ranging from 0.09 µg/L to 50.88 µg/L. From site 1, Co concentrations showed a range of 0.20–18.84 µg/L and a mean of 2.19 ± 4.43 µg/L. Although the mean Co concentration from site 2 was 1.08 ± 1.34 µg/L, it was recorded as the lowest when compared to sites 1 and 3. However, the mean concentrations for all the sites were higher than the recommended limit for human exposure as set by the WHO. No significant difference was observed in the blood concentrations of Co in participants from the sites in this study (*p* > 0.05). The consumption of beer may be a source of high levels of Co recorded from the blood of participants as they were observed to be drinking during sampling from both sites 1 and 2. Although participants from site 3 were not observed to be drinking beer during the sampling visits, they do consume other alcoholic beverages. Studies also showed that individuals who consume high volumes of alcohol, especially beer with added cobalt chloride (CoCl_2_) or cobalt sulphate (CoSO_4_), a foam stabilizer, may suffer from cardiomyopathy [60,61,62]. Reports show that Mn and Co concentrations in beer samples ranged from 25.29 to 228.60 μg/L and 0.16 to 0.56 μg/L, respectively [63,64]. Therefore, the consumption of alcoholic beverages, which include beer, may also contribute to the high levels of Co observed in all the sites in the study. Furthermore, the length of stay of obese females living around site 3 shows that individuals who lived in the area for more than 10 years recorded the lowest levels of blood Co compared to those who have stayed in the area for less than 10 years (Figure 3), therefore suggesting exposure from previous residential areas. Cameán et al. [65] have also reported that Co and Mn are critical metals that can be found in high concentrations in coal-derived sources, such as coal refuse, coal combustion products, and coal acid mine drainage. Eren et al. [66] reported that Co has a concentration of 25 ppm in the Earth’s crust, which in addition to coal burning through mining and industrial activities, has resulted in the high blood Co concentrations observed from all the sites in this study. A report by Talan et al. [67] shows that high levels of Co has been associated with autism in children.

**Figure 4:**
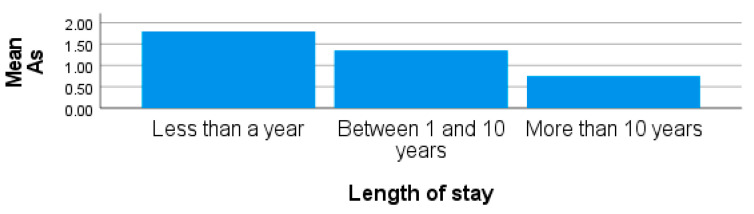
Showing blood As for length of stay in obese females living around site 3.

The blood As concentrations from participants residing in site 3 were the highest, with minimum and maximum values of 0.70 µg/L and 3.43 µg/L, respectively, and a mean of 1.37 ± 0.67 µg/L. From site 1, the mean concentrations of As in the blood were 0.84 ± 0.41 µg/L and ranged from 0.27 µg/L to 2.20 µg/L (Table 1). From the results, it was observed that participants from sites 1 and 2 recorded lower mean concentrations. However, the concentrations of As from some participants from both sites were higher than the WHO recommended limit of 1.00 µg/L. There was no significant difference observed in the concentrations of blood As in participants from the sites in this study (*p* > 0.05). The high blood As recorded from site 3 could be historical traces of their previous and current living area (Figure 4). Furthermore, the burning of coal has been associated with high levels As [42,43], and other sources of As in the environment are anthropogenic in nature, as well as due to lifestyle habits of active or secondary smoking [26,56,68]. Lion and Olowoyo [24] have also reported that humans living around mining areas can be exposed to high levels of As, Mn, Cr, and Cd through inhalation. Arsenic is carcinogenic and can cause cancer in different parts of the body and chronic diseases, such as diabetes and hypertension [32,69].

**Figure 5:**
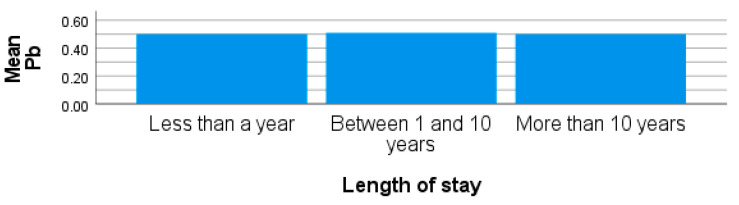
Showing blood Pb by length of stay in obese females living around site 3.

From site 2, the mean blood Pb concentration was higher than those recorded for participants from sites 1 and 3 with a mean, standard deviation and range of 19.41 ± 11.84 µg/L (6.70–60.20 µg/L). The concentration of Pb from site 1 was also higher than the recommended limit of 0.80 µg/L, with a mean of 6.70 ± 3.40 µg/L and a range of 5.00–18.40 µg/L. Not all the participants had blood Pb higher than the recommended limit. Although the mean blood Pb concentrations from some participants from site 3 (0.51 ± 0.05 range 0.50–0.78 µg/L) were lower than the recommended limit, but with a caution as trace metals could bioaccumulate. There was a significant difference observed in the concentrations of blood Pb in participants from sites 1, site 2, and site 3 in this study (*p* < 0.01). Figure 5 shows that the levels of blood Pb recorded from all the participants from different sites in the study may be due to exposure to lead and not necessarily associated with the areas used for this study. The ban on unleaded petrol only came into effect in South Africa in 2005, and previous studies have shown the ability of Pb to remain in the environment for a prolonged period of time due to its non-biodegradable nature. Some studies have also reported high levels of Pb [70,71]. The report showed that once Pb is absorbed in the blood, it has the ability to cause anemia by decreasing the number of red blood cells in the body. The results obtained from participants residing in site 2 were comparable with that of Shekar et al. [72], where the highest concentrations of blood Pb from the exposed group were 45.43 ± 6.93 μg/L, which exceeds the recommended limit of 08.0 μg/L for human blood.

**Figure 6:**
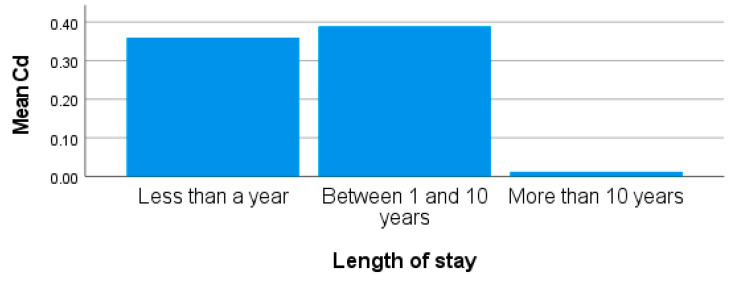
Showing blood Cd by length of stay in obese females living around site 3.

Blood Cd from this study recorded the lowest value of all the trace metals in the participants’ blood. The mean concentration of Cd in the blood was lower than the recommended limit of 1.12 µg/L from all the participants. However, higher than the recommended limit of Cd in blood were recorded from some females residing in site 3. The length of stay of obese females living around site 3 shows that individuals who lived in the area for more than 10 years recorded lower levels of blood Cd than those who had stayed in the area for less than 10 years (Figure 6). The length of stay shows that participants may have been exposed to high levels of Cd from other sources. The recorded concentrations for Cd were 0.38 ± 0.55 µg/L (0.01–2.28 µg/L) from site 3. There was no significant difference observed in the concentrations of blood Cd in participants from the sites in this study (*p* > 0.05). Though lower concentrations of As, Pb, and Cd (Table 1) were recorded in the blood of some participants, there is a need for close monitoring to reduce the effects of exposure on human health. High levels of Cd in the blood may affect the respiratory, renal, and skeletal systems. These high levels can lead to renal and tubular dysfunction and an increased risk of cardiovascular disease, peripheral arterial disease, heart failure, myocardial dysfunction, and stroke [73,74]. This study is similar to that conducted by [20], where they reported an increase in the concentrations of Pb, Mn, and Cd in the blood of participants exposed to trace metal pollutants. Furthermore, the study by Devoy et al. [75] also reported higher concentrations of mean blood Cd and Pb exceeding the permissible limits of 1.12 μg/L and 0.80 μg/L, set by WHO, respectively. Their findings further show that high levels of these metals can be attributed to several factors, such as smoking, place of residence, and intake of some food.

The participants in our study reported frequent headaches and dizziness as some of the major health problems encountered. This phenomenon is similar to those reported in the study [59]. Despite the fact that there are limited studies on occupational exposures to Mn, the study of [76], a study of haemolysis of particulate matter (PM) on red blood cells from a coal-burning lung cancer epidemic area, the report showed that Mn had a significant positive correlation with the oxidative capacity of PM in coal-burning environments, and this is a usual practice from some of the participants in our study. Furthermore, the report by [77] shows that Mn concentrations in blood cells increase as the dose of external exposure increases. Generally, the high levels of Mn in South African soils have previously been reported in the literature [78]. We do not know clearly if some of the participants in our study practice geophagia; however, this is a common practice in South Africa, and this may be responsible for the increase in the value of Mn recorded in the blood of some of the participants [79].

## 4. Conclusions

To the best of our knowledge, this is the first study to explore exposure to trace metals from obese females living in informal settlements around industrial areas. This study can assist in forming a baseline for future research by monitoring and assessing human health risks in these areas. From this study, it was evident that mean Mn levels in blood from participants in site 1 were higher than the other two sites, while the mean blood Pb in site 2 was also higher than in other areas. However, it is important to note that some of the participants from site 3 (Industrial area) also showed high levels of blood Cr, As, Co, and Cd. The results of participants from site 3 also showed that these participants resided in some of the areas that were used in this study, which included industrial activities such as mining areas and coal-fired power stations. The results of the study showed that industrial activities may not be the only route for human exposure to trace metals; as noted in this study, lifestyle may also play a major role in the blood levels of these toxic trace metals. Levels of some trace metals recorded from some of the participants in this study may require further intervention due to reports received from the participants and from previous studies. An increase in the levels of these trace metals in the blood may pose a health risk to obese female participants. Future studies should be geared towards examining the sources of blood trace metals recorded from participants in this study, especially from site 3, in this study.

## Figures and Tables

**Table 1:** Trace metals in blood of obese females living around study sites in µg/L.

Metals	Concentrations [µg/L; x̅ ± SD (Range)]			
	Site 1	Site 2	Site 3	WHO
Mn *	12.97 ± 6.04 (6.79–33.99)	11.41 ± 4.35 (5.50–24.40)	11.37 ± 4.73 (4.72–22.95)	12.60
Cr	3.70 ± 4.11 (0.41–19.01)	ND	6.16 ± 6.30 (1.58–29.29)	0.23
Co	2.19 ± 4.43 (0.20–18.84)	1.08 ± 1.34 (0.02–5.50)	2.40 ± 8.92 (0.09–50.88)	0.30
As	0.84 ± 0.41 (0.27–2.20)	0.95 ± 0.99 (0.03–5.70)	1.37 ± 0.67 (0.70–3.43)	1.00
Cd	0.20 ± 0.20 (0.02–1.10)	0.25 ± 0.26 (0.01–0.90)	0.38± 0.55 (0.01–2.28)	1.12
Pb *	6.70 ± 3.40 (5.00–18.40)	19.41 ± 11.84 (6.70–60.20)	0.51± 0.05 (0.50–0.78)	0.80

(Site 1: industrial area, Site 2: industrial area, and Site 3: residential area) (* *p* < 0.01).

## Data Availability

The data are not publicly available due to ethical, privacy and legal issues.
